# Effects of Inclusive Leadership on Quality of Care: The Mediating Role of Psychological Safety Climate and Perceived Workgroup Inclusion

**DOI:** 10.3390/healthcare10112258

**Published:** 2022-11-11

**Authors:** Momna Yousaf, Muhammad Majid Khan, Adil Tahir Paracha

**Affiliations:** Department of Management Sciences, COMSATS University Islamabad, Islamabad 44000, Pakistan

**Keywords:** professional diversity, intergroup context, leadership, inclusion, quality of care

## Abstract

Purpose: The aim of the study is to investigate the multilevel effects of the inclusiveness of workgroup leaders on quality of care by intervening through a “psychological safety climate” at the group level and “perceived workgroup inclusion” at the individual level within professionally diverse workgroups of healthcare professionals. Material and Methods: Data are collected from 305 healthcare professionals nested in 61 workgroups and 305 patients treated by the same workgroups working in public-sector hospitals in Pakistan. Hypothesized relationships are tested through multilevel analyses using Mplus 7. Results: The results of the study show that inclusive leadership can enhance the quality of care delivered by multiprofessional workgroups of healthcare professionals through perceived workgroup inclusion. Further, the psychological safety climate does not mediate the relationship between inclusive leadership and quality of care individually, but it transmits the effects of inclusive leadership through perceived workgroup inclusion on quality of care. Conclusion: The results of the study suggest that the inclusiveness of workgroup leaders, the psychological safety climate, and perceived workgroup inclusion can create safe and inclusive interpersonal mechanisms that play a key role in transmitting the positive effects of inclusive leadership on quality of care.

## 1. Introduction

The quest of healthcare organizations to deliver a good quality of care with an acute shortage of healthcare professionals and fewer resources in hand necessitates the exploration of more effective ways to organize and manage the existing workforce. Quality of care is considered a performance indicator for healthcare professionals working in groups [1]. These workgroups, whether working in conventional formations or controlled combinations of professional silos, become multiprofessional in nature and ineluctably acquire the characteristics of professional diversity [2] due to the presence of two or more individuals with professionally diverse backgrounds [3]. Based on this premise, the quality of care is mainly attributed to the communication of different care-related perspectives and informational resources among these individuals [4,5]. Conversely, the context of multiprofessional workgroups, especially in traditional settings, is based on the hierarchical order of authority [6] and is more pronounced between doctors and nurses [7]. The deeply rooted status hierarchy [8] and strong professional identities [9] impede the process of communication and make it challenging for workgroup leaders to integrate different care-related perspectives of healthcare professionals for the effective delivery of care [10]. Consequently, there are calls in the literature to investigate the role of leadership in the unique context of multiprofessional or professionally diverse workgroups of healthcare professionals for the enhancement of the quality of care [11,12,13].

Previous research argued that to strive for favorable leadership behaviors, it is necessary to identify salient diversity-generating processes within a group [14,15]. According to the categorization elaboration model (CEM) [14], these processes are classified as ‘information elaboration’ (implying the exchange of information, communication, discussion, and the integration of different perspectives) and ‘subgroup categorization’ (implying the individual’s propensity to categorize themselves and similar others into “in-group” and dissimilar others into “out-group”). Within multiprofessional workgroups, the intertwining effects of diverse professional identities (e.g., doctors and nurses) and well-entrenched status hierarchies yield both high intragroup similarities and high intergroup differences due to differences in knowledge and educational backgrounds. These differences build an individual’s subjective frame of reference (his or her beliefs, expectations, and stereotypes) about his/her professional identity, inducing the formation and salience of subgroups, and disrupts inter-subgroup relations [2,14,16]. It also reduces the motivation and ability of individuals to communicate or share information [17,18] and results in poor quality of care.

Inclusive leadership was found to be helpful in integrating different professional perspectives [9,19]. It was established that inclusive leadership could be positively associated with employee quality engagement behaviors [19], the enhanced performance of professionally diverse teams [9], and increased unit performance [20]. However, these studies built leadership perspectives without focusing on salient diversity-generating processes within workgroups [15] and paid little attention to how inclusive leadership could improve QOC within a professionally diverse and status-oriented context of multiprofessional workgroups of healthcare professionals.

Numerous studies have established a reliable association between inclusive leadership and workgroup inclusion [21]. The current literature confirms that workgroup inclusion can reduce subgroup categorization [22,23], and the perception of having an insider status (a related concept of inclusion) by members can enhance the QOC delivered by groups (wards) [24]. The perceived workgroup inclusion of individuals can act as a possible mechanism through which the inclusiveness of a workgroup leader can enhance the quality of care delivered by multiprofessional workgroups of healthcare professionals. Similarly, the literature has also established the association between inclusive leadership and the psychological safety climate [20]. Workgroup inclusion as a result of inclusive leadership creates a psychological safety climate, which helps in reducing individuals’ defensiveness and in motivating them to share information without fear of adverse consequences [25] regardless of their status in the hierarchy and professional identity. Psychological safety is also recognized as a key theoretical factor of the individual perception of inclusion [26]. Surprisingly, the impact of the psychological safety climate at the group level is somewhat an under-researched area [27], and is not linked with the quality of care. Recently, Durand et al. suggested investigating the effects of a psychologically safe climate on individuals in the context of healthcare [28].

Keeping in view the above discussion, this study is an attempt to build and test a multilevel inclusive leadership perspective with an eye on the emergence of salient diversity-generating processes. It may, more precisely, provide novel contributions to the existing literature by identifying that a shared perception of inclusive leadership can enhance the quality of care delivered by multiprofessional workgroups of healthcare professionals through the psychological safety climate and perceived workgroup inclusion.

## 2. Literature Review

Leadership can have a tremendous impact in healthcare settings. For example, it affects group practices [11,24,29,30] and contributes to safe and quality patient care [31]. In particular, inclusive leadership deviates from the traditional approach and focuses on relational, communicative, and participative work environments [32] through “exhibiting words and deeds that invite and appreciate others’ contributions” [19]. However, recently, Weiss et al. [7] argued that this conceptualization of the inclusiveness of workgroup leaders may not work for every group member that belongs to a different status and professional identity in the same way, because it focuses more on the leader to value the uniqueness of individuals to overcome status differences without identifying any concrete behaviors of the leader. Thus, the present study argues that a shared perception of a leader’s inclusiveness can enhance the quality of care by minimizing the circumstances that supplement the salience of subgroup categorization. The shared perception of group members is grounded in the idea that the leader acts in a similar manner with all group members [33] and leadership is oriented towards social interaction, as continuous social interaction influences group members’ collective interpretation and makes it more homogeneous [34,35]. The leader’s openness, accessibility, and availability [36] has the potential to build a shared perception of inclusiveness of the workgroup leader across status lines of professions and help alleviate subgroup categorization.

A widely accepted idea in the literature is that quality of care is a multidimensional concept with several interrelated dimensions [37] grounded in patients’ experiences of the care delivered by multiple healthcare professionals [38]. Thus, quality of care is defined as the performance outcome of multiprofessional workgroups of healthcare professionals. The previous literature also identified that quality of care is achieved when healthcare professionals from different disciplines work together [39].

Similarly, Shore and colleagues [40] summed up the previous literature in light of the optimal distinctive theory [41]. They described perceived workgroup inclusion as being based on balancing the individual needs for belongingness and uniqueness. According to Chung et al. [21], perceived workgroup inclusion is a key variable for enhancing the performance of group members. It can also reduce the effects of subgroup categorization and engage group members in information sharing and open communication behaviors [42], where group members feel like an important part of the group.

The psychological safety climate has been referred to as a shared perception among members that the group is safe to take interpersonal risks and encourages the exchange of different task-related perspectives and knowledge without the fear of any repercussions or backlash from the members of the group [27,43]. It creates a perception among group members that each one of them can speak up without worrying about their respective statuses or professional norms, and it helps group members in sharing their professional insights more effectively [8]. The perception of such a safe climate within the group also results in the reporting of treatment errors [44]. Such an environment increases interpersonal communication and results in improved healthcare outcomes [11,45,46,47].

### 2.1. Inclusive Leadership and Quality of Care

The leader’s openness, accessibility, and availability [36] has the potential to build a shared perception of inclusiveness for workgroup leaders across status lines of professions. From the social information processing perspective, the openness of a workgroup leader generates informational cues about the willingness of the work group leader to listen to members’ ideas and be receptive to their input, and, hence, members become more likely to contribute and communicate within the group [18,48,49]. The availability and accessibility of a leader generates informational cues, which encourage group members to actively consult with the leader and increase the instances of social interactions [50]. These behaviors enhance the level of proximity (referred to as “the extent to which one could be exposed to social information in a given social system” [51]) and can frame a collective mindset or shared perception about the leader’s inclusion [52], which motivates the members to share information and communicate across the status lines and work together as a group rather than working in subgroups or professional silos. This collective stance reduces the perception of subgroup categorization. These information-sharing behaviors exhibited by all professionals within a workgroup enable it to effectively identify and address all aspects of patient requirements and needs to, subsequently, bring accuracy in the diagnosis and treatment and reduce the risk of medical errors through the use of diverse expertise, which, ultimately, enhances the quality of care [11,28,30,53,54]. Keeping in view the above discussion, the following hypothesis is proposed:

**Hypothesis** **1.** *Inclusive leadership can be positively associated with the quality of care delivered by multiprofessional workgroups of healthcare professionals.*

### 2.2. Inclusive Leadership, Perceived Workgroup Inclusion, and Quality of Care

The previous literature identified the link between inclusive leadership and positive employee attitudes, such as job satisfaction [55], team identity [9], and workgroup inclusion [21], which are related to enhanced work performance [56]. Similarly, a shared perception of the inclusiveness of workgroup leaders may positively influence the quality of care by fostering the perception of the inclusiveness of workgroup members. From the social information perspective [57,58], the shared perception of inclusive leadership generates informational cues that foster a workgroup environment, which enhances the tendency of individuals to perceive members of other professions as part of the “in-group” [7,59]. According to the common bound theory, this view of inclusion reduces status differences and breaks “out-group” boundaries, and individual members consider themselves full members of the group. This phenomenon also reduces the salience of subgroup categorization. It also increases the motivation of group members to share knowledge across professional boundaries [5] and stimulates the process of communication. The perception of inclusion also improves the commitment of group members, which, in turn, leads them to work harder to improve group performance [60]. Workgroup inclusion also encourages a group-directed helping attitude [61] that compels group members to engage in collaborative work and frequent communication. Evidence also suggests that the quality of care delivered by those groups (wards) is better than others, providing a strong perception of insider status (being part of the group) [24]. Taking it all together, the present study proposes the following hypothesis:

**Hypothesis** **2.** *Inclusive leadership can be positively associated with quality of care through its influence on perceived workgroup inclusion.*

### 2.3. Inclusive Leadership, Psychological Safety Climate, and Quality of Care

Leadership can positively affect work outcomes through its effects on the organizational climate [62,63]. According to the functionalist perspective, leaders can shape the work environment by influencing it, which, ultimately, affects performance [64,65]. It also accelerates the process of information-sharing within the group, improves the quality of care delivered by a diverse group of healthcare professionals through better interpersonal communication and knowledge sharing [66,67], and, more importantly, allows members to raise their concerns regarding work-related issues, such as adverse events [43,44]. On the other hand, recently, Knippenberg et al. [18] identified that the psychological safety of an individual employee is not a sufficient condition for information elaboration within groups. Concerning this, the psychological safety climate can be a possible condition for enhancing the information elaboration process within a group, because it can be a psychological mechanism that fosters a shared environment that motivates individual members to communicate and discuss within the group. The psychological safety climate provides an arena where group members can develop a perception that the group has acceptability of their views, which can accelerate the process of information sharing. Inclusive behaviors of leaders are essential for creating a psychological safety climate within groups for improving quality of care [45], because they engage with staff from different disciplines and hierarchal levels, and encourage true participation within healthcare settings [2,66]. It enables them to elaborate on care-related information within the group. The reason is that they can discuss errors or unexpected outcomes freely and do not feel hesitant in seeking feedback and giving their unique perspective, which leads to the effective delivery of care. Taking it all together, this study proposes the following hypothesis:

**Hypothesis** **3.** *Inclusive leadership can be positively associated with quality of care through its influence on the psychological safety climate.*

### 2.4. Psychological Safety Climate, Perceived Workgroup Inclusion, and Quality of Care

The psychological safety climate enhances group-level positive outcomes, such as group learning behavior and group performance [68], and reduces errors, which can increase quality of care [69]. The psychological safety climate of a workgroup enables and supports members to be part of critical organizational processes [25] and enhances their perception of inclusion, which positively affects their work performance. Previous studies also suggest that a psychological safety climate facilitates the communication process within a group via the exchange of perspectives without any psychological distress [66] and, hence, enriches the perception of inclusion as group members process more information and show better performance [20]. The psychological safety climate enhances interpersonal communication and knowledge sharing within a group [67,68], which is an important component to balancing the need for belongingness and uniqueness [18]. It reduces the salience of subgroup categorization. Therefore, regardless of their status within the group, group members accomplish their tasks effectively, provide solutions to problems, and help other group members complete their jobs successfully. The psychological safety climate, thus, satisfies employees’ socioemotional need for inclusion, and employees who feel more included are also more committed [56], more satisfied with their jobs [69], and perform better [21], all of which directly or indirectly impacts the QOC provided to the patients in healthcare organizations [70]. Therefore, the study proposes the following hypothesis:

**Hypothesis** **4.** *The psychological safety climate can be positively associated with quality of care through its influence on perceived workgroup inclusion.*

The present study argues that the shared perception of leader’s inclusiveness enhances the psychological safety climate, and the psychological safety climate enhances an individual’s perception of workgroup inclusion, which, in turn, enhances quality of care. In combination, these arguments suggest the final hypothesis.

**Hypothesis** **5.** *Inclusive leadership can be indirectly associated with the quality of care through its influence on the psychological safety climate and perceived workgroup inclusion.*

### 2.5. Theoretical Framework and Model

**Figure 1:**
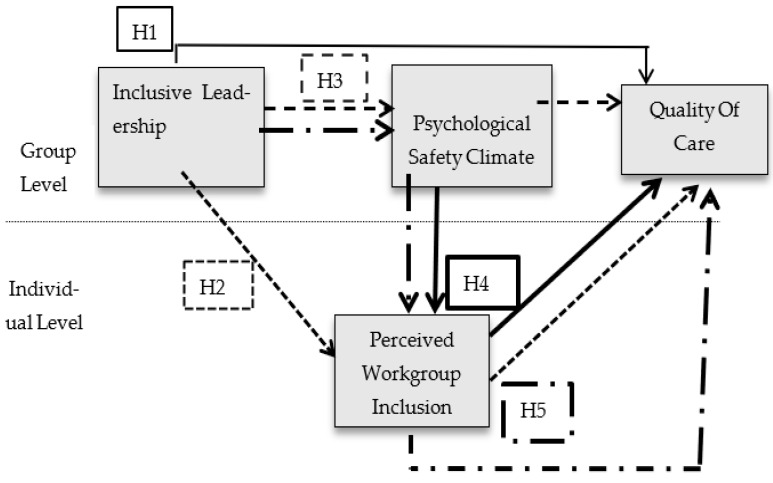
Multilevel hypothesized model. The dashed lines in Figure 1 separate the group-level variables (i.e., that is, inclusive leadership, psychological safety climate, and quality of care) and individual-level variables (i.e., perceived workgroup inclusion).

The conceptual model elaborates on the relationship between variables. In the present study, inclusive leadership was taken as the independent variable, quality of care as the dependent variable, the psychological safety climate as the group level mediator, and perceived workgroup inclusion was treated as the individual mediator. The categorization elaboration model, social information processing theory, common bound theory, and social exchange theory underpin the research model. The research model was conceptualized within the broader framework of the categorization elaboration model, which sheds light on the context of the workgroups of healthcare professionals and helps in identifying the salient diversity-generating processes. The basic idea of the social information processing theory is that a shared perception of inclusive leadership can generate inclusive informational cues, which build a climate of psychological safety at the group level and perceived workgroup inclusion. The common bound theory establishes that perceived workgroup inclusion extends the in-group boundaries and enhances communication and information-sharing, resulting in a better quality of care. According to the social exchange theory, a psychological safety climate builds high-quality social exchange relationships among the members of a group [8,43]. Such relationships involve mutual concern and support that creates a sense of obligation to reciprocate, and results in an enhanced quality of care (see Figure 1).

## 3. Material and Methods

### 3.1. Sample and Procedure

The data were collected from 70 workgroups working in accredited departments (accredited by the College of Physicians and Surgeons, Pakistan) of eleven public teaching hospitals providing inpatient care, located in Pakistan, and from patients treated by the same workgroups through a self-administered questionnaire. To identify workgroups with the above-mentioned characteristics, hospitals were visited and members of workgroups and patients treated by the same workgroups were identified through detailed discussions with ward managers, senior residents, and nurses. As per the previous convention of aggregating measures at the group level [71], the study chose workgroups with a minimum of five group members. The sample did not include workgroups and patients from emergency, pediatric, psychiatric, intensive care units (ICUs), and coronary care units (CCUs), because patients of these wards were not in a condition to answer about the quality of care provided to them. The study only included those patients who were admitted in the same ward for at least 1 whole day and were more than 18 years of age. The purpose and method of data collection was explained to the healthcare professionals and patients to obtain their consent. Even after giving consent, patients could terminate the data collection process at any time they wished to. Each construct was measured using different scales and compounded in the survey under a separate head to acquire more accurate and unbiased responses (i.e., psychological separation [72]).

A total of 305 patients (76% response rate) and 305 healthcare professionals (76% response rate) participated in the research, including 85 nurses, 73 senior registrars, 100 junior registrars, 43 house officers, and 7 technicians. Complete data were obtained from 61 workgroups. The average response rate was 76%. For healthcare professionals, 53.8% of participants was female and 46.2% male. In total, 33.1% of this sample reported ages of between 20 and 29, 61% was between 30 and 39, and 5.9% between 40 and 49. The largest category of healthcare professional samples constituted those with a tenure of between 3 and 5 years (43.6%). The second largest frequency of professionals fell in the tenure category of 6–10 years (29.5%). In total, 23.9% belonged to the tenure category of 1–2 years. The tenure category of above 10 years was constituted of the smallest number of healthcare professionals (3%). In the case of the patients, 56% was female and 44% male. The self-reported ages of 24.3% of the patients were between 18 and 29, 41.3% were between the ages of 30 and 39, 26.9% were between the ages of 40 and 49, 5.9% between the ages of 50 and 60, and only 1.6% were those with ages of 60 or above. Most of the patients had a higher secondary school certification (41.3%), 34.4% held graduate degrees, 27.2% either had a secondary school certification (10 years of schooling) or below, 3.6% were postgraduates, and only 3% held professional degrees. Of this sample, 48% patients were hospitalized for 1–2 days, 47% for 3–4 days, and only 5% for 5–6 days.

### 3.2. Measures

To reduce the common method bias, measures were obtained from the workgroups of healthcare professionals and patients simultaneously [73]. Inclusive leadership was conceptualized as a group-level construct and measured via a survey of group members using an adapted version of the scale developed by Carmeli et al. [36]. It measured openness (3 items), availability (4 items), and accessibility (2 items) on a five-point Likert scale of 1 = not at all and 5 = to a large extent. Recently, this measure was used to assess the inclusiveness of leaders at a group level [74]. Keeping in view the suggestion of González-Romá [75] about multilevel studies, a clear reference of the group level construct was used in each item of the construct by adding the work group. The sample items included “leader of my workgroup is open to discuss the organization’s desired goals and new ways to achieve them”.

The psychological safety climate was conceptualized and measured via an adapted version of the 7-item scale developed by Edmondson [43]. Responses were determined on a five-point Likert scale ranging from one (not at all) to five (to a large extent). One sample of the items included “If you make a mistake in this workgroup, it is often held against you”.

The scale for the perceived workgroup inclusion consisted of 10 items recently developed by Chung et al. [21]. It measures belongingness (five items) and uniqueness (five items) on a five-point Likert scale, ranging from strongly disagree (=1) to strongly agree (=5). The phrasing of the items included first-person singulars such as ‘I’ and ‘my’ in order to clearly project that the item was related to the respondents’ own perception of inclusion. A sample item read ‘I am treated as a valued member of my work group’.

An adapted 4-items scale developed by Dagger et al. [38] was used to measure patients’ ratings of quality of care delivered by the workgroup of healthcare professionals. It is a self-administered measure and ranged from 1 (strongly disagree) to 5 (strongly agree). A sample item read ‘The overall quality of the care provided at this Ward is excellent’.

## 4. Results

The data in this study were analyzed by using Mplus version 7.1 [76]. To examine the hypotheses, a multilevel path analysis was conducted using the same software. As respondents of this study were clustered into sixty-one groups, this design suggested that observations were dependent on team membership. To test this assumption, Muthén’s intraclass correlation was computed for the endogenous factor of the quality of care. The intraclass correlation was high enough to justify running multilevel analyses (ICCM = 0.683). Therefore, a two-level CFA model was estimated to study the psychometric properties of the model and instruments.

### 4.1. Measurement Properties

Initially, the overall validity of the single level, four-factor CFA model was weak (χ^2^ = 966.420; df = 399; *p* = 0.000; RMSEA = 0.068; CFI = 0.920; TLI = 0.913; SRMR = 0.044) following the suggestion of modifying indices (M.I. = 75.803; M.I. = 41.373) for the residual of the third and fourth items of the quality-of-care instrument and residuals of the seventh and eighth items of workgroup inclusion instruments, respectively. Covarying these error terms improved the global fit indices to an acceptable level (χ^2^ = 859.233; dF = 397; *p* = 0.000; RMSEA = 0.062; CFI = 0.935; TLI = 0.929; SRMR = 0.041). By extending the model to a two-level measurement model, acceptable global fit indices were obtained (χ^2^ = 1172.948; df = 796; *p* = 0.000; RMSEA = 0.039; CFI = 0.909; TLI = 0.900; SRMRw = 0.065; SRMRb = 0.058). This model was comparatively better than its alternative models, as shown in Table 1. Concerns of the common method bias also dissipated when poor global fit indices were obtained for a two-level, one-factor measurement model (χ^2^ = 1713.016; df = 808; *p* = 0.000; RMSEA = 0.061; CFI = 0.781; TLI = 0.764; SRMRw = 0.096; SRMRb = 0.094). After establishing the overall validity of the two-level, four-factor model, the convergent and discriminant validities and interitem reliability of the instruments were evaluated at a between-group level.

Table 2 and Table 3 show that the convergent validity, discriminant validity, and reliability of all four instruments were supported at the between-group level, as determined by the greater than 0.50 average variance extracted (AVE) of the individual latent variables, significant chi-square difference test (∆χ^2^) of pairs of latent variables, and greater than 0.70 construct reliability (Hair et al. 2019). The bivariant Pearson correlation coefficient among the four latent variables showed that inclusive leadership was positively correlated with the psychological safety climate, workgroup inclusion, and quality of care (r = 0.945; *p* = 0.000; r = 0.917; *p* = 0.000; r = 0.819; *p* = 0.000). Then, the psychological safety climate was positively correlated with workgroup inclusion and quality of care (r = 0.944; *p* = 0.000; r = 0.832; *p* = 0.000). Additionally, workgroup inclusion was positively correlated with quality of care (r = 0.868, *p* = 0.000).

### 4.2. Composite

High-factor loadings (λ = 0.963 to 0.993) and substantial shared variance (31.5%) stimulated the development of an index of quality of care by averaging the four items (α = 0.898). High-factor loadings (λ = 0.846 to 0.999) and substantial shared variance (39.4%) inspired the creation of an index of workgroup inclusion by taking the average of the ten items (α = 0.928). High-factor loadings (λ = 0.893 to 0.977) and substantial shared variance (45.8%) stimulated the creation of an index of the psychological safety climate by averaging the seven items (α = 0.900). High-factor loadings (λ = 0.962 to 0.994) and substantial shared variance (40.4%) motivated the creation of an index of inclusive leadership by averaging the nine items (α = 0.946). These indices were used in further analyses.

### 4.3. Data Aggregation

Table 4 shows average item ICC (1), average item ICC (2), and average group rwg(j) for the indices of quality of care, the psychological safety climate, and inclusive leadership. The average items for the ICC (1) of quality of care, psychological safety climate, and inclusive leadership were greater than or equal to 0.420, 0.579, and 0.489, respectively. The average items for the ICC (2) of quality of care, psychological safety climate, and inclusive leadership were greater than or equal to 0.782, 0.870, and 0.826, respectively. Similarly, average group values for the rwg(j) of quality of care, psychological safety climate, and inclusive leadership were greater than or equal to 0.948, 0.968, and 0.978, respectively. These values highlighted that team membership was affecting the ratings of respondents, there were reliable differences in the mean ratings across teams, and there was a strong agreement among respondents. Thus, it was appropriate to aggregate the quality of care, psychological safety climate, and inclusive leadership. After the aggregation of these variables, this study carried out a multilevel path analysis to test a series of hypotheses, including the relationship of aggregated inclusive leadership to aggregated quality of care via the serial mediation of an aggregated psychological safety climate and unaggregated workgroup inclusion.

### 4.4. Hypotheses Testing

**Figure 2:**
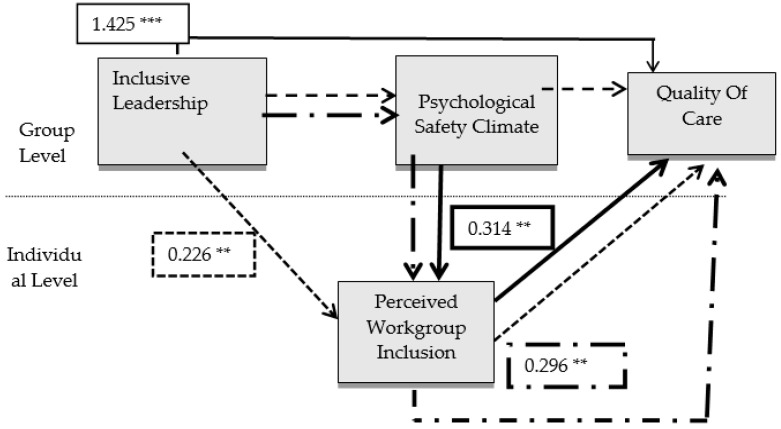
Multilevel mediating effect model. Level 1—individuals; Level 2—groups. Only significant coefficients are shown; ** *p* < 0.05, *** *p* < 0.001.

Part A of Table 5 displays that a positive association between inclusive leadership and the perceived quality of care (Hypothesis 1) was supported (γ = 1.593; *p* = 0.000). Table 5, part B, shows that the indirect effect of inclusive leadership on the quality of care via perceived workgroup inclusion (Hypothesis 2) was supported (γ = 0.336; Bayesian CI = 0.079 to 0.683). The indirect effect of inclusive leadership on quality of care via the psychological safety climate (Hypothesis 3) was not supported (γ = 0.075; Bayesian CI = −0.425 to 0.512). The indirect effect of the psychological safety climate on perceived quality of care via perceived workgroup inclusion (Hypothesis 4) was also supported (γ = 0.223; Bayesian CI = 0.086 to 0.570). Lastly, the serial indirect effect of inclusive leadership on the *p* quality of care via the psychological safety climate and perceived workgroup inclusion (Hypothesis 5) was supported (γ = 0.276; Bayesian CI = 0.093 to 0.591). Figure 2 also represents the significant indirect effects.

## 5. Discussion

The current study examined the impact of inclusive leadership on the quality of care delivered by professionally diverse workgroups of healthcare professionals. In addition, the mediating role of the psychological safety climate and perceived inclusion was hypothesized and investigated. Overall, the findings supported the conceptualized model and revealed the mechanism through which the shared perception of inclusive leadership could improve the psychological safety climate and perceived workgroup inclusion and patient-related performance outcomes, i.e., quality of care. Specifically, the study demonstrated that inclusive leadership could enhance the quality of care delivered by multiprofessional workgroups of healthcare professionals. These study results were consistent and extended the current conversation about inclusive leadership and its impact on healthcare outcomes [54]. The findings indicated that QOC was achievable and associated with the extent to which group members developed perceptions of the inclusiveness of their workgroup leader. It was also found that inclusive leadership had an indirect effect on the quality of care through the mediating effect of perceived workgroup inclusion. These findings complemented and were consistent with the existing research on leadership and healthcare outcomes [24,31]. This finding indicated that the inclusiveness of workgroup leaders could reduce the adverse effects of intergroup bias [32]. In such an environment, group members considered themselves as part of the group or included. Such situations lessen the perceptions of communication barriers among nurses and doctors [31] and diminish the concern that care-related information shared by low-status professionals (such as junior doctors and nurses) are not considered openly. This phenomenon could most likely increase the motivation of every individual to communicate and build strong relationships among nurses and doctors, leading to the effective delivery of care. On the other hand, the empirical test of the indirect effect of inclusive leadership on quality of care via the psychological safety climate demonstrated no support. These results reinforced the observation of van Knippenberg et al. [18], that not only individuals’ perceptions of psychological safety, but also the climate of psychological safety was not a sufficient condition to improve group outcomes. In addition, the status difference among the group members created a difference in the perception of psychological safety within subgroups [45]. That is why a safe interpersonal climate alone may not affect the communication or process of information elaboration to the extent that it can improve group outcomes. It is because of a strong in-group or out-group sense that does not allow group members to break the out-group boundaries. As a result, some group members may not feel that the group climate is safe to take interpersonal risks, and they withhold care-related information, which does not enhance the effective delivery of care. Interestingly, the results of the study identified a positive and significant influence of psychological safety climate on quality of care through perceived workgroup inclusion. This finding was consistent and extended past research [21,26,40] by providing empirical evidence. These findings suggested that within a psychological safety climate, members think that the group cannot reject, punish, or embarrass anyone for giving their contribution, which enhances mutual respect for each other’s competence [27,43]. In this sense, the psychological safety climate addressed the barriers to interactions among professional hierarchies within workgroups of healthcare professionals [8] and built a perception of more supportive interpersonal relationships within workgroups of healthcare professionals and individuals who felt more comfortable in sharing their unique perspectives, which enhanced their feelings of inclusion. The feeling of inclusion provided the comfort needed for group members to avoid their individual differences in work processes, which, ultimately, resulted in quality of care.

Similarly, serial mediation was also supported between inclusive leadership, the psychological safety climate, perceived workgroup inclusion, and quality of care. These results established that, in multiprofessional or professionally diverse workgroups, where it is difficult to flatten the status hierarchy among subgroups, inclusive leaders can create an environment that harnesses the PSC, and the compound influence of these factors builds a positive group climate in which group members (even lower-status members) feel valued, included, and recognized for her/his competence [9]. This environment can help in reducing the effects of subgroup categorization and enhance information elaboration within groups to perform QOC. These findings also highlighted that the individual perception of inclusion within multiprofessional workgroups is key to translate the effects of inclusive leadership and the psychological safety climate into quality of care. Similarly, these results were also consistent with the argument of Shore et al. [41], who considered the inclusiveness of workgroup leaders and the psychological safety climate to be necessary conditions to foster the perception of inclusion within a context where status and categorization is more salient. The factors build an environment where all group members, regardless of their status and professional identities, are deemed part of the group, which enhances the quality of care [54].

In terms of theoretical implications, this study added to the limited research on inclusive leadership and quality of care in the context of multiprofessional workgroups of healthcare professionals. Specifically, it capitalized on the basic tenants of the categorization elaboration model [14] and social information processing theory [57] to understand the salient professional diversity-generating processes for attaining benefits from professional diversity and identifying direct and indirect antecedents of the quality of care. In addition, the study added to the small, yet emerging, body of research that examines inclusive leadership as a group-level phenomenon. Our study postulated the notion that a leader’s openness, availability, and accessibility during interactions enable members to develop a collective perception regarding the extent to which the leader is inclusive and values their perspectives, which, in turn, enables them to communicate and share knowledge freely by easily approaching the leader for discussion and consultation about the ongoing process of care. The study tested the proposed model in the context of healthcare, in which communication and information-sharing are largely impeded by the presence of status silos among professions, holding a particular significance in impacting patients and staff alike [28,77]. Furthermore, the present study measured the quality of care delivered by workgroups of healthcare professionals working in public-sector hospitals from the patients’ perspective, providing a first-hand account of the delivery of care. Finally, instead of focusing on the direct effects of diversity on group processes and outcomes, the study contributed to group diversity research by identifying that the workgroups operated with different levels of statuses among professions.

From a practical standpoint, this study suggested that healthcare managers can utilize the true potential of employees through the enactment of their own behaviors, which can enhance communication and the integration of unshared information, which then, ultimately, results in an enhanced quality of care.

## 6. Conclusions

The study aimed to investigate the role of workgroup leaders to help healthcare organizations to meet this challenge. This research integrated the theories of leadership, diversity, and inclusion to build a multilevel model of the effects of inclusive leaders, the psychological safety climate, and perceived workgroup inclusion on the quality of care delivered by multiprofessional workgroups of healthcare professionals. The results obtained from the MSEM analyses identified that inclusive leaders and the psychological safety climate can build safe and inclusive interpersonal mechanisms for enhancing the perception of work group inclusion, which plays a key role in transmitting the positive effects of inclusive leadership on to quality of care. These findings could be used to develop strategies to improve and support the inclusive behaviors of workgroup leaders for enhancing QOC. There were some limitations to the present study that could be translated into opportunities for future research. First of all, the study was cross-sectional in nature, and gave only an initial basis of a conceptual model of the mechanisms operating the link between inclusive leadership and quality of care. Further studies should replicate our findings with research designs that allow for a more robust testing of causal relationships. Further, the quality of care was measured through the patients’ perceptions. Future research should measure it with more objective measures of healthcare quality. Another limitation to consider was the generalizability of the sample. Although it was taken from public teaching hospitals and teaching hospitals can share most characteristics with one another, they probably still differ from private teaching hospitals. Therefore, future research can focus on private-sector hospitals to enhance the generalizability of the research. Furthermore, the sample used in this study was moderately diverse. Future studies could replicate this model in a more diverse setting.

## Figures and Tables

**Table 1:** Alternate two-level measurement models.

Alternate Models ^t^	χ^2^	df	RMSEA	CFI	TLI	SRMR_w_	SRMR_b_
1. Four-factor model at group and individual levels (IL, PSC, WI, QOC)	1172.948 ***	796	0.039	0.909	0.900	0.065	0.058
2. Three-factor model at group and individual levels (Combined IL and PSC items as one factor; WI and QOC as other two factors)	1230.545 ***	802	0.042	0.896	0.888	0.070	0.066
3. Three-factor model at group and individual levels (combined PSC and WI items as one factor; IL and QOC as other two factors)	1271.922 ***	802	0.044	0.886	0.877	0.072	0.061
4. Three-factor model at group and individual levels (combined WI and QOC items as one factor; IL and PSC as other two factors)	1484.956 ***	802	0.053	0.835	0.821	0.089	0.082
5. Two-factor model at group and individual levels (combined items of IL, PSC, and WI as first factor, and QOC as the other factor)	1382.375 ***	806	0.048	0.861	0.849	0.081	0.075
6. Two-factor model at group and individual levels (combined IL and PSC items as one factor; WI and QOC as the other factor)	1550.794 ***	806	0.055	0.820	0.805	0.092	0.086
7. One-factor model at group and individual levels	1713.016 ***	808	0.061	0.781	0.764	0.096	0.094

Note: Nj—61. Ni—305. t—in every two-level model, each factor was composed of two subfactors; that is, one at within-group level and the other at between-group level. IL—inclusive leadership. PSC—psychological safety climate. WI—workgroup inclusion. QOC—quality of care. *** *p* < 0.001.

**Table 2:** Discriminant validity.

Factor Pairs	2 Factor MCFA χ^2^ (df)	1 Factor MCFA χ^2^ (df)	∆χ^2^ (∆df)	Discriminant Validity
1. Inclusive Leadership and Psychological Safety Climate	369.046 *** (206)	425.951 *** (208)	56.905 ** (2)	Supported
2. Inclusive Leadership and Workgroup Inclusion	571.796 *** (302)	791.931 *** (304)	220.135 ** (2)	Supported
3. Inclusive Leadership and Quality of Care	246.926 *** (128)	699.888 *** (130)	452.962 ** (2)	Supported
4. Psychological Safety Climate and Workgroup Inclusion	452.950 *** (236)	534.726 *** (238)	81.776 ** (2)	Supported
5. Psychological Safety Climate and Quality of Care	181.298 *** (86)	278.108 *** (88)	96.810 ** (2)	Supported
6. Workgroup Inclusion and Quality of Care	303.979 *** (152)	548.144 *** (154)	244.165 ** (2)	Supported

Note: Nj—61. Ni—305. ** *p* < 0.05. *** *p* < 0.001.

**Table 3:** Convergent validity, interfactor correlations, and construct reliability.

Factors	AVE	Standard Deviation	1	2	3	4
1. Inclusive Leadership	0.950	0.635	(0.994)			
2. Psychological Safety Climate	0.879	0.676	0.945 ***	(0.981)		
3. Workgroup Inclusion	0.898	0.627	0.917 ***	0.944 ***	(0.989)	
4. Quality of Care	0.978	0.561	0.819 ***	0.832 ***	0.868 ***	(0.994)

Note: Nj—61. Ni—305. AVE—average variance extracted. *** *p* < 0.001. Parentheses contain construct reliabilities.

**Table 4:** Overall aggregation assessment.

Factors	ICC (1)	ICC (2)	r_WG(J)_
Quality of Care	0.420	0.782	0.948
Psychological Safety Climate	0.579	0.870	0.968
Inclusive Leadership	0.489	0.826	0.978

ICC—intraclass correlation.

**Table 5:** Hypotheses testing.

Structural Relations	Unstandardized Structural Coefficient γ	*p*	Bayes 95% Credibility Interval
Part A: Direct Associations 1. IL_A → QOC_A	1.425	0.000	-
Part B: Indirect Associations 2. IL_A → PSC_A → QOC_A	0.023	0.888	−0.366 to 0.442
3. IL_A → WGI → QOC_A	0.226	0.031	0.060 to 0.517
4. PSC_A → WGI → QOC_A	0.314	0.016	0.097 to 0.562
5. IL_A → PSC_A → WGI → QOC_A	0.296	0.018	0.086 to 0.538

Note: Nj—61. Ni—305. Bayes method was used to generate 95% credibility intervals. IL_A—aggregated inclusive leadership. PSC_A—aggregated psychological safety climate. WGI—workgroup inclusion. QOC_A—aggregated quality of care.

## Data Availability

The data presented in this study can be provided upon request to the corresponding author. For ethical reasons, these data cannot be made public.
